# Solitary Fibrous Tumor

**DOI:** 10.3390/cancers16213573

**Published:** 2024-10-23

**Authors:** Bahil Ghanim

**Affiliations:** 1 Karl Landsteiner University of Health Sciences, Dr. Karl-Dorrek-Straße 30, 3500 Krems, Austria; bahil.ghanim@kl.ac.at; 2 Department of General and Thoracic Surgery, University Hospital Krems, Mitterweg 10, 3500 Krems, Austria

Solitary fibrous tumor (SFT) is an orphan disease of mesenchymal origin. The tumor can occur anywhere in the human body but is most frequently found in the chest. Surgery remains the standard of care in all SFTs. However, even completely resected disease can recur many years after surgery and its clinical behavior is unpredictable [1]. Genetic characterization using NAB2-STAT6 fusion has helped to better define and thus understand the disease for over a decade [2]. In addition, some risk stratification models have been developed and validated to estimate the clinical outcome [3]. 

Regarding therapy, surgery remains the cornerstone in SFT management but systemic treatment (including antiangiogenetic therapy) as well as radiotherapy have been suggested to improve survival and quality of life, especially in advanced stages of the disease [4]. Despite the current advantages of non-surgical SFT therapy, there is still an urgent need to enhance our clinical and biological understanding of this rare malignancy.

The present Special Issue aims to improve—for patients as well as for physicians —the frustrating situation in which an established and effective therapy and follow-up strategies are still lacking, and focuses on providing an overview of the current standard of care by contributing three review articles. 

In addition, seven research articles enhance the knowledge regarding risk stratification and novel treatment approaches. On the one hand, a better risk stratification is urgently needed to establish follow-up strategies to treat this orphan disease in a clinically meaningful manner. On the other hand, an effective therapy that could provide an alternative to surgery is of interest for non-resectable patients, as well as in the setting of multimodal therapy before and/or after surgery. We hope that this Special Issue will help to improve the outcome of this rare and challenging disease. 
